# Fetal intestinal fibroblasts respond to insulin-like growth factor (IGF)-II better than adult intestinal fibroblasts

**DOI:** 10.1186/1471-213X-6-4

**Published:** 2006-01-27

**Authors:** Mark R Corkins, Michael J Fillenwarth

**Affiliations:** 1 Division of Gastroenterology, Hepatology and Nutrition, James Whitcomb Riley Hospital for Children, Indiana University School of Medicine, Indianapolis, IN, USA, 46202

## Abstract

**Background:**

We compared IGF responses of fetal and adult intestinal fibroblasts to identify a developmental difference in the IGF-axis. Intestinal fibroblasts were isolated from maternal and fetal jejunum. Media was conditioned at confluence and one week afterwards. The proliferative response at confluence to 5 nM IGF-I or -II was compared.

**Results:**

There were no significant differences in IGFBP expression at confluence. Post-confluence, fetal fibroblasts had no significant changes in IGFBP-2 and IGFBP-3 expression. Post-confluent maternal fibroblasts had increased IGFBP-3 levels that were significant compared to the fetal fibroblasts. IGF-I increased in post-confluent fetal fibroblasts, while in maternal fibroblasts it decreased (p < 0.001). IGF-II secretion decreased significantly in post-confluent maternal fibroblasts (p < 0.05). Maternal fibroblasts proliferated more with IGF-I than IGF-II (p < 0.001). Fetal fibroblasts responded to IGF-II slightly better than IGF-I and significantly greater than maternal cells (p < 0.001).

**Conclusion:**

Fetal intestinal fibroblasts respond to IGF-II with greater proliferation and do not have the increased IGFBPs seen post-confluence in adult intestinal fibroblasts.

## Background

Insulin-like growth factors are peptide growth factors and have been found to be powerful mitogens for intestinal epithelial cells with modulation by six specific IGF binding proteins (IGFBPs) [1,2]. The IGFBP species have been shown to inhibit or potentiate the proliferative response to IGFs, depending on the cell type and culture conditions [1]. The primary IGF found in serum during prenatal development is IGF-II [3]. IGF-II mRNA levels in the human duodenum were also found to be five times higher in the fetus than in the child [4].

The IGFBP-3 which is normally a negative modulator of growth also has a developmental pattern of expression. A study that examined 18 normal fibroblast cell lines created from subjects ranging from fetal to 76 years of age found progressively increasing amounts of IGFBP-3 production with age [5]. This direct correlation of donor age and IGFBP-3 levels was consistent during all phases of fibroblast cell growth [5]. Fetal sheep have high circulating IGFBP-2 serum levels that peak at approximately 70% (105 days) of gestation, then begin to fall [6]. In studies measuring tissue specific mRNA levels, fetal tissue IGFBP-2 follows a pattern similar to serum in fetal sheep [7]. Another study, comparing post-natal intestinal and adult total intestinal mRNA for the IGFBPs, found higher mRNA post-natal levels of IGFBPs-3 through 6 that fell with age. [8].

The fibroblasts IGF axis is important in the intestine because of the paracrine effect on the intestinal mucosa. A dual culture model found that intestinal epithelial cells grown on intestinal fibroblasts had greater proliferation when stimulated by the paracrine effect of fibroblast produced IGF-II [9]. A transgenic mouse model with IGF-I overexpression coupled with a mesenchymal promoter was found to have increased IGF-I overexpression in the gastrointestinal lamina propria this resulted in a mucosa with increased DNA and protein content manifested by increased crypt cell mitosis and sucrase activity [10].

This study was designed to compare the regulation of the IGF-axis and the IGF responses of fetal and adult intestinal fibroblasts. The hypothesis was that fetal small intestinal fibroblasts would have a higher production and respond better to IGF-II than adult intestinal fibroblasts. We also expected to find the IGFBPs levels present to decrease to allow increased proliferation in the fetal intestinal fibroblasts.

## Results

### Protein assay of conditioned medium sample

The media conditioned by the cultured maternal or fetal intestinal fibroblasts was collected at confluence and post-confluence. The conditioned media was stored at -20°C until analysis. The measured protein concentrations did not vary significantly for the maternal or fetal fibroblasts comparing confluence or post-confluence. There was also no significant difference between maternal or fetal conditioned media at either time point measured (range 1.10 to 1.16 mg/ml).

### IGF-I and IGF-II expression

The conditioned media from the maternal and fetal intestinal fibroblasts was assayed for IGF concentration by radioimmunoassays. The maternal and fetal intestinal fibroblasts produced primarily IGF-II. At confluence, the fetal fibroblasts produced greater concentrations of IGF-I and IGF-II than maternal fibroblasts, but this did not achieve statistical significance (8.4 ± 1.7 vs. 5.1 ± .03 ng/ml IGF-I, p = 0.5) (40 ± 3.0 vs. 30 ± 1.3 ng/ml IGF-II, p = 0.08).

**Figure 1:**
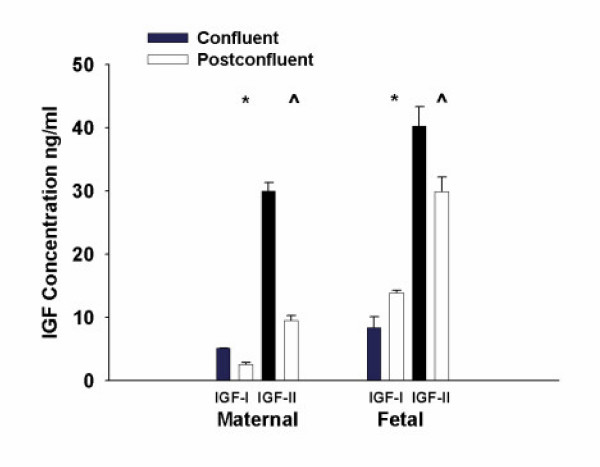
Concentrations of IGF-I and IGF-II in the conditioned media. The maternal and fetal intestinal fibroblasts produced primarily IGF-II. In this representative experiment the mean and SEM data represents the pooled data from triplicate samples from the three cell lines. The fetal fibroblasts produced greater concentrations of IGF-I and IGF-II than maternal fibroblasts but did not achieve statistical significance (8.4 ± 1.7 vs. 5.1 ± 0.03 ng/ml IGF-I, p = 0.53 and 40.3 ± 3.0 vs. 29.99 ± 1.3 ng/ml IGF-II, p = 0.08). The one week post-confluence fetal fibroblasts had increased IGF-I secretion (13.9 ± 0.4), while that of the maternal fibroblasts decreased (2.7 ± 0.3,* p < 0.001). The IGF-II secretion was decreased slightly one week post-confluence in fetal fibroblasts (29.9 ± 2.3) and significantly in maternal fibroblasts (to 9.5 ± 0.8, ^p < 0.05).

One week post-confluent fetal fibroblasts had increased IGF-I secretion, while that of the maternal fibroblasts decreased (13.9 ± 0.4 versus 2.7 ± 0.3 ng/ml IGF-I, p < 0.001). The IGF-II secretion was decreased slightly one week post-confluence in fetal fibroblasts, and it was significantly decreased in maternal fibroblasts (29.9 ± 2.3 versus 9.5 ± 0.8 ng/mL IGF-II, p < .05). (Figure 1)

### Ligand blotting for IGFBP expression

Ligand blotting utilizes the high affinity of the IGFBPs for the IGFs to detect the presence of IGFBP species present in the conditioned media. Three IGFBP bands were seen consistently with relative molecular weights that have been characterized for ovine IGFBPs-3 (42–50 kDa doublet), -2 (33 kDa) and -4 (24 kDa) [7,11,12]. Immunoblotting was performed and verified that the 33 kDa and 24 kDa bands were IGFBP-2 and -4 respectively (data not shown). The fibroblasts produced primarily IGFBP-2 in both maternal and fetal cell lines. At confluence, there were no significant differences in the expression of the IGFBPs between the fetal and maternal fibroblasts (IGFBP-3: fetal 114 520 ± 7 500 versus maternal 140 250 ± 15 240 AIU, p = 0.147; IGFBP-2: fetal 463 130 ± 61 590 versus maternal 362 170 ± 54 830 AIU, p = 0.415; IGFBP-4: fetal 244 360 ± 59 510 versus maternal 128 900 ± 19 710 AIU, p = 0.330).

**Figure 2:**
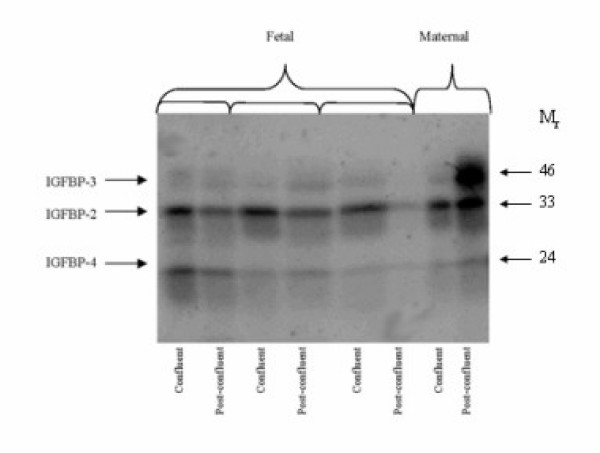
**Ligand blot comparison of IGFBP expression**. Three IGFBP bands were seen consistently with relative molecular weights that have been characterized for ovine IGFBPs-3 (42–50 kDa doublet), -2 (33 kDa) and -4 (24 kDa) [7,11,12]. The fibroblasts produced primarily IGFBP-2 in both maternal and fetal cell lines. The IGFBP levels did not significantly change one week post-confluence in fetal cells compared to maternal cells; which had a general increase in IGFBP expression.

Fetal fibroblasts had non-significant changes in expression of IGFBP-3 one-week post-confluence (114 520 ± 7 500 versus 122 170 ± 17 880 AIU, p = 0.759) and nearly significant reductions in IGFBP-2 (463 130 ± 61 590 versus 323 940 ± 52 590 AIU, p = 0.098). In contrast, maternal fibroblasts post-confluence had nearly significant increased IGFBP-2 (362 170 ± 54 830 versus 841 450 ± 117 030 AIU, p = 0.082) and significantly increased IGFBP-3 (140 250 ± 15 240 versus 1 123 800 ± 17 820 AIU, p = 0.002). There were no significant changes in any of the IGFBP-4 levels measured. The increases in IGFBP-2 and IGFBP-3 in the post-confluent maternal fibroblasts were significant when compared to the post-confluent fetal fibroblasts (IGFBP-3: p < 0.001 and IGFBP-2: p = 0.003). (Figure 2)

### Fibroblast response to IGF-I and IGF-II

**Figure 3:**
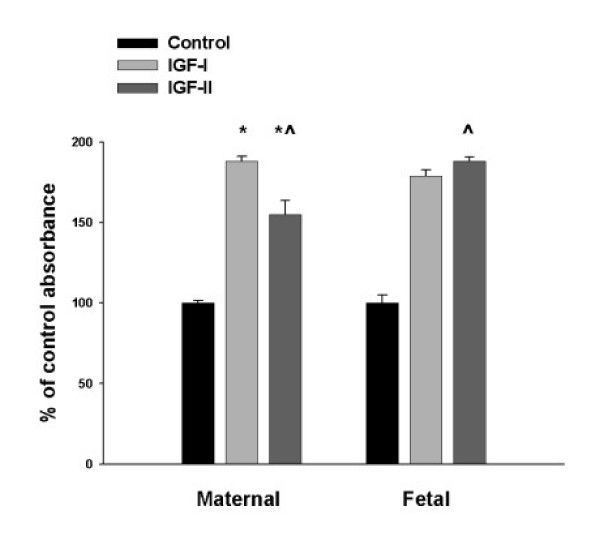
**The fibroblast response to IGF-I and IGF-II**. The MTT assay demonstrated that all populations of fibroblasts responded to IGF treatment with statistically significant (p < 0.001) greater cell numbers. In this representative experiment the mean and SEM data represents the pooled data from duplicate samples from the three cell lines. Maternal fibroblasts responded with greater cell numbers for IGF-I than IGF-II compared to controls (88% vs 55% increase,* p < 0.001). Fetal fibroblasts responded to IGF-II better than IGF-I, although not significantly, and at the same rate as maternal fibroblasts responded to IGF-I (88% vs 79%, p = 0.34). The amount of increase in the fetal cells in response to IGF-II was significantly greater that the amount of increase seen in the maternal cells (^p < 0.001). Error bars are standard error of the mean calculated from the original absorbance data and divided by the mean absorbance of the controls to create a percent value.

Maternal and fetal fibroblasts were grown to confluence and then treated with 5 nM recombinant human IGF-I or IGF-II. The MTT assay demonstrated that all populations of fibroblasts responded to IGF treatment with statistically significant (p < 0.001) greater cell numbers. A percentage was calculated for graphic presentation, for statistical calculations the actual measured absorbance at 570 nm was utilized. Triplicate wells of each experimental condition was undertaken for each of the cell lines. Maternal fibroblasts responded with greater cell numbers for IGF-I than IGF-II compared to controls (88% vs 55% increase, p < 0.001). Fetal fibroblasts responded to IGF-II better than IGF-I, although not significantly, and at the same rate that maternal fibroblasts responded to IGF-I (88% vs 79%, p = 0.34). The amount of increase in the fetal cells in response to IGF-II was significantly greater that the amount of increase seen in the maternal cells (p < 0.001). (Figure 3)

## Discussion

The primary finding from our studies is the increased responsiveness of the fetal intestinal fibroblasts to IGF-II compared to the adult intestinal fibroblasts. The adult intestinal fibroblasts responded to IGF-II with only a 55% increase in the cell numbers compared to controls. The fetal intestinal fibroblasts had the maximum response to IGF-II with an 88% increase in cell numbers. Interestingly, this is the same rate at which the adult intestinal fibroblasts responded to IGF-I. Circulating IGF-I levels are low in fetal sheep and increase throughout gestation, while circulating IGF-II and small intestine IGF-II mRNA levels are much higher and increase to a peak late in gestation [6,7]. The primary prenatal IGF in mice and humans is IGF-II as well, but in adults the primary circulating IGF is IGF-I [1,3]. Therefore, our study suggests that the intestinal fibroblast proliferation response to the IGFs is developmentally regulated for the maximum response dependent on developmental stage of the individual.

Comparing the IGF concentrations used to stimulate fibroblast proliferation to physiologic conditions, Bloomfield et al. reported IGF-I concentrations in sheep amniotic fluid of 0.25 nM at 110–112 days gestation [13]. A paper from Blahovec et al. reported that the amniotic fluid at 70 days gestation had concentrations that were roughly1.28 nM IGF-I and 16.4 nM IGF-II; subsequent western blotting for IGF-II throughout gestation showed increasing concentrations until it peaked between 106 and 120 days gestation [14]. Studies have shown poor absorption of enteral IGFs after birth but there are several studies in fetal animals indicating significant absorption of ingested IGFs [15,16]. The 5 nM concentration utilized in these experiments correlates well with the reported IGF-I and -II concentrations in porcine milk (ovine has not been reported) during the immediate post-partum period [17]. The fetal intestinal fibroblast would be expected to be stimulated by approximately this concentration of IGFs from both serum and luminal sources.

Both maternal and fetal intestinal fibroblasts produced primarily IGF-II. The fetal intestinal fibroblasts maintained a relatively high level of IGF expression even when post-confluent while the maternal intestinal fibroblasts had significant decreases in IGF levels. This is consistent with human studies which found greater levels of IGF-II mRNA in the duodenum in fetal intestine compared with children and adults [4]. This study also found the total intestinal mRNA for IGF-I was higher than for IGF-II in the adult [4]. A study characterizing the IGF production in a human colon fibroblast cell line and primary culture adult small intestinal fibroblasts found significant IGF-II and low levels of IGF-I mRNA [9]. Our cell culture data that intestinal fibroblasts produce primarily IGF-II and that the greatest level of production is by the fetal intestinal fibroblasts is consistent with the literature mRNA studies. The IGFBP findings were not what we hypothesized. The fetal intestinal fibroblasts had no significant changes when post-confluent. This is in comparison to the highly significant increase in IGFBP-3 seen in the post-confluent maternal intestinal fibroblasts. Fetal rat lung fibroblasts plated near confluence had increased levels of IGFBPs -2 and -3 and stable IGFBP-4 levels after 24 hours of culture in serum-free media, similar to our conditions [18]. An older study that examined IGFBP production by developmental stage; found that fetal sheep skin fibroblasts had relatively high levels of IGFBP production [19]. The IGFBPs produced by the fetal skin fibroblasts were reported by relative molecular weight to be consistent with IGFBPs -2 and -4 compared to low levels of IGFBP-3 [19]. The concentration of the IGFBP with a relative molecular weight consistent with IGFBP-3 had a marked increase in the perinatal period [19]. A study by Goldstein et al that examined IGFBP-3 in 18 normal fibroblast cell lines (skin and lung) found increasing IGFBP-3 levels that correlated with the donors chronological age [5]. This study found increased IGFBP-3 levels at all age levels with increased culture time [5]. The significant increase in production of IGFBP-3 by the maternal intestinal fibroblasts is very consistent with the pattern seen in these other fibroblast cell lines [5]. The lack of a significant change in IGFBP levels in the fetal intestinal fibroblasts is in contrast to the increased levels seen in the other studies of fetal fibroblasts [5,18]. This lack of increase could be speculated as significant finding because it would allow continued stimulation of the fetal intestinal fibroblasts by the IGFs present.

The fetal intestinal fibroblasts are important in the development of the intestinal mucosa. A transgenic mouse model with a mesenchymal promoter linked to an IGF-I gene found increased IGF-I overexpression in the gastrointestinal lamina propria resulted in increased crypt cell mitosis and sucrase activity [10]. This evidence would suggest a role for paracrine stimulation by the fetal intestinal fibroblast produced IGFs on the intestinal mucosa. The persistent production of IGFs by the fetal intestinal fibroblasts suggests the developmental regulation of IGF expression for paracrine stimulation of the fetal intestinal mucosa.

## Conclusion

In this study, we always found a higher production of both IGFs by fetal intestinal fibroblasts compared to maternal intestinal fibroblasts. We also demonstrated increased proliferation by fetal compared to adult intestinal fibroblasts in response to IGF-II, the primary circulating IGF in the fetus.

## Methods

### Materials

Cell culture media, sera and reagents were obtained from Life Technologies, Grand Island, NY. Tissue culture flasks and six-well plates were from Falcon (Becton Dickinson, Lincoln Park, NJ). Recombinant human [3-^125^I-iodotyrosyl] IGF-I and -II were provided by M. H. Niedenthal (Lilly Research Laboratories, Indianapolis, IN). The IGF-II antibody was purchased from Upstate Biotechnology (Lake Placid, New York). BA 75 nitrocellulose was purchased from Schleicher and Schuell (Keene, NH). Unless stated, all other chemicals were obtained from Sigma (St. Louis, MO) or Fisher (Pittsburgh, PA).

### Cell culture

The protocol was approved by the Indiana University Animal Care and Use Committee. This project utilized pregnant domestic farm sheep cared for in an American Association for Laboratory Animal Care-certified facility. Fetal sheep were delivered at 130 days gestation (85% of term, equivalent to human 34 weeks gestation). Fetal and maternal jejunum were harvested.

The intestine was minced and incubated in tissue culture dishes. After 72 h, fibroblasts had migrated onto the dish and residual tissue was removed. This is similar to a technique utilized by Fowlkes and Freeman to study sheep skin fibroblasts [19]. Gastrointestinal epithelial and endothelial cells do not easily grow under primary cell culture conditions. This is the reason that there are limited numbers of GI cell lines that are not derived from cancer cell lines available. Fibroblasts quickly take to primary cell culture and proliferate rapidly. After one passage, no evidence of anything but fibroblasts was in evidence microscopically. The primary culture fibroblasts were used with a maximum of five passages before the studies were performed. The fibroblasts were cultured in standard DMEM media with 10% fetal bovine serum. The cells were grown in culture to visual confluence and maintained in culture one week past confluence. The cells at confluence and one week post-confluence were allowed to condition serum-free media containing 0.1% BSA for 24 hours. This media was harvested and stored at -20°C. Three separate fetal-maternal experiments were used to create three intestinal fibroblast cell cultures.

Maternal and fetal fibroblasts were plated equally in 12-well plates and grown to confluence. This time point was chosen to evaluate the response to the IGFs while the fibroblasts were still actively proliferating but experiments could visually be matched at equal cell density. Microscopically, the fibroblasts would continue to proliferate until the culture plate surface was saturated with fibroblasts. Six wells of each plate of the various fibroblast cell lines were treated with control serum-free media and the other three with 5 nM recombinant human IGF-I or IGF-II. This dosage was utilized since it was above the minimal dose to see a response in a previous study comparing young and senescent fibroblasts [20]. The change in cell numbers was compared by MTT assay after 24 hours. Absorbance measurements were obtained from three wells for each of the three fetal and maternal cell cultures. These were used for significance calculations.

### Protein assay

The protein content of the conditioned medium was measured utilizing the BCA reagent according to the manufacturer's guidelines (Pierce, Rockford, IL). The reaction of protein in an alkaline environment results in the reduction of copper to a cuprous cation. The cuprous cation reacts with the bicinchoninic acid to produce a colorimetric change that is linear over a wide range. Bovine serum albumin was utilized to create a standard curve for the optical density at a wavelength of 570 nm with the BCA assay. The protein concentrations of duplicate samples from each of the three different cell lines were determined from the standard curve.

### IGF radioimmunoassay

The IGFs were extracted from the conditioned media by aliquoting the samples into 0.8 M formic acid/0.5% Tween 20. Acetone was added to precipitate the binding proteins. The samples were then centrifuged at 40°C for 20 minutes. The extracted samples were incubated with competing ^125^I labelled rhIGF-I or rhIGF-II (tracer) and antibody (polyclonal rabbit anti-rhIGF-I or anti-rhIGF-II monoclonal antibody) (Amano International Enzyme, Troy, VA). The bound and free fractions were separated by precipitation of the bound IGF with 10% polyethylene glycol and a second antibody against the primary antibody. The greater the concentration of IGF in the sample the lower the amount of the radioactive labelled tracer that was bound by the antibody. Three separate culture plates for each of the three cell lines created were analyzed. IGF concentrations of samples were estimated from a standard curve of reference ovine IGF-I or IGF-II purified from serum. The radioactivity in the precipitate was measured in a gamma counter and the resulting data was analyzed by a weighted four parameter logistic algorithm [21].

### Ligand blotting

Ligand blot analysis was performed as described by Hossenlopp et al [22] and modified by McCusker et al [23]. The media samples were electrophoresed in 12.5% polyacrylamide gels in the presence of 0.1% SDS under non-reducing conditions. Following electroblotting onto 0.05 μm BA-85 nitrocellulose, the blots were probed with ^125^I-IGF-II. The IGF-II binds with high affinity to the IGFBPs separated by electrophoresis. Previous studies have demonstrated that the amount of ^125^I-IGF-II radioactive binding is linear with the IGFBP concentrations in the respective bands [24]. Signal intensities for radioactive bands on each blot were quantified by use of a PhosphorImager (Molecular Dynamics, Sunnyvale, CA) with the results reported as Arbitrary Intensity Units or AIUs. Three IGFBP bands were seen consistently with relative molecular weights that have been characterized for ovine IGFBPs-3 (42–50 kDa doublet), -2 (33 kDa) and -4 (24 kDa) [7,11,12]. Samples from each of the separate cell lines underwent the ligand blotting

### Western blotting

Conditioned media samples and a conditioned media from a human cell culture known to produce IGFBP-2 as well standard IGFBP-4 were electrophoresed in a 15% polyacrylamide gel in the presence of SDS under non-reducing conditions. Following electroblotting onto Immobilon P (Millipore, Inc., Bedford, MS), the blots underwent western blotting with either anti-human IGFBP-4 antibody (Austral Biologicals, San Ramon, CA) or an anti-bovine IGFBP-2 antibody (Upstate Biotechnology, Lake Placid, NY). The blot was labelled with ^125^I-protein A and exposed to film for autoradiography. The 42–50 kDa band was assumed to be ovine IGFBP-3 based on relative molecular weight as there is no antibody available that reacts with ovine IGFBP-3.

### MTT assay

The cell numbers were compared using the technique described by Denizot and Lang [25]. The media is harvested and the cells are incubated with a solution of the MTT (3- [4,5-dimethlythiazol-2-yl]-2,5 diphenyl tetrazolium bromide) salt for 3 h at 37°C. Living cells reduce this compound to a blue formazan product that can be measured in a spectrophotometer. There is a linear relationship between the number of cells and the amount of the formazan product produced. The MTT solution is poured off and the plate rinsed with saline. After drying, the plate is treated with isopropanol to dissolve the reaction product. An aliquot is then measured for absorbance at 570 nm in the spectrophotometer. Duplicate samples of each of the three cell lines were analyzed.

### Analysis and statistics

Values for each group are expressed as mean ± the standard error of the mean (SEM). This was calculated from the pooled results of the duplicate or triplicate assays for each assay (n = 6 or 9). Every experiment was performed at least twice to confirm the results. Statistical analysis was performed utilizing SigmaStat software from SPSS, Inc (Chicago, IL). Analysis between two groups was done by unpaired two-tailed Student's t-test. Analyses between multiple groups were determined by one-way ANOVA utilizing All Pairwise Multiple Comparison Procedures (Tukey test) with 95% confidence intervals.

## List of abbreviations

IGF, insulin-like growth factors; IGFBP, insulin-like growth factor binding proteins; AIU, arbitrary intensity units; SEM, standard error of the mean

## Authors' contributions

MRC designed the studies, performed the ligand blotting, did the statistical analysis and performed the cell culture work. MF performed all of the assays except the ligand blotting. Both authors have read and approved the final manuscript.
